# Increased *in vitro* antimicrobial resistance of *Mycoplasma pneumoniae* isolates obtained from children in Beijing, China, in 2023

**DOI:** 10.3389/fcimb.2024.1478087

**Published:** 2024-12-20

**Authors:** Xinyu Jia, Yujie Chen, Yagang Gao, Xue Ren, Bing Du, Hanqing Zhao, Yanling Feng, Guanhua Xue, Jinghua Cui, Lin Gan, Junxia Feng, Zheng Fan, Tongtong Fu, Ziying Xu, Zihui Yu, Yang Yang, Shuo Zhao, Lijuan Huang, Yuehua Ke, Chuanhe Liu, Chao Yan, Jing Yuan

**Affiliations:** ^1^ Department of Bacteriology, Capital Institute of Pediatrics, Beijing, China; ^2^ Department of Allergy, Children’s Hospital of Capital Institute of Pediatrics, Beijing, China

**Keywords:** *Mycoplasma pneumoniae*, antimicrobial susceptibility, macrolide resistance, molecular characteristics, children

## Abstract

**Introduction:**

*Mycoplasma pneumoniae* (*M. pneumoniae*), a common pathogen of community-acquired pneumonia in school-age children and adolescents, can cause epidemics worldwide. In late 2023, the incidence of *M. pneumoniae* infection among children reached a high level.

**Methods:**

We investigated the *in vitro* antimicrobial susceptibility of 62 *M. pneumoniae* isolates obtained from children with pneumonia in Beijing between 2021 and 2023, and analyzed the correlation of antimicrobial susceptibility with molecular characteristics of isolates and clinical manifestations of patients.

**Results:**

The resistance rates of *M. pneumoniae* isolates against erythromycin and azithromycin were both 100% (62/62). The minimum inhibitory concentration (MIC) of acetylspiramycin (16-membered macrolides) was lower than that of erythromycin and azithromycin. The MIC of azithromycin in 2023 was notably higher compared to 2021 and 2022. No resistance to tetracycline and levofloxacin was observed. Genotypes P1 type 1 and P1 type 2 were identified in 74.2% and 25.8% of isolates, and M4-5-7-2 (61.3%) and M3-5-6-2 (22.6%) were predominant multi-locus variable-number tandem-repeat analysis (MLVA) types. The A2063G mutation was present in all isolates (100%). Among the patients, 45/59 cases (76.3%) had severe *M. pneumoniae* pneumonia, and 14/59 cases (23.7%) presented co-infection. The duration of fever was 12 days (1-30 days) and the fever duration after initiation of macrolide antibiotics treatment was 8 days (1-22 days).

**Discussion:**

Our study showed that macrolide-resistant *M. pneumoniae* (MRMP) with high *in vitro* antimicrobial resistance level may be the causative factor of the *M. pneumoniae* epidemic in late 2023 in Beijing, China. It is urgent to pay more attention to MRMP and the antibiotics choose.

## 1 Introduction

*Mycoplasmas* are the smallest and simplest self-replicating prokaryotic organisms that often parasitize mucus surfaces in the respiratory and genitourinary tracts (Benedetti et al., 2020). *Mycoplasma pneumoniae* (*M. pneumoniae*) is one of the most common pathogens responsible for community-acquired pneumonia, especially in children and adolescents (Su et al., 2021). In the USA, an estimated 2 million cases of *M. pneumoniae* occur annually, resulting in approximately 100,000 hospitalizations, with school-age children and adolescents being the most commonly affected (Waites et al., 2019). *M. pneumoniae* produces a variety of virulence factors, such as membrane lipoproteins, polysaccharides, and invasive nucleases, which induce proinflammatory cytokines, lymphocyte activation, airway pathology, and dysfunction, and can lead to a variety of pulmonary disorders (Ramasamy et al., 2018; Luo et al., 2021).

The epidemiologic cycle of *M. pneumoniae* infection is 3 to 7 years. In late 2019 and early 2020, *M. pneumoniae* simultaneously became endemic in several European and Asian countries. The epidemiologic surveillance of acute respiratory infections in China showed that the *M. pneumoniae* infection rate ranked third among childhood pneumonia cases and first among adult pneumonia cases (Li et al., 2021). Interestingly, the positive detection rate of *M. pneumoniae* RNA decreased significantly from 40% to 10% during the pandemic period of COVID-19 (Li et al., 2023). However, in 2023, *M. pneumoniae* infections rapidly increased to 4.12% in European countries, and the positive detection rate in China rose to 61.1% (Meyer Sauteur and Beeton, 2023; Gong et al., 2024; Yan et al., 2024).

Because *Mycoplasma* has no cell wall, the first-choice treatment for *M. pneumoniae* infection is macrolide antibiotics which act on bacterial ribosomes and inhibit protein synthesis, along with tetracyclines and fluoroquinolones (Waites and Talkington, 2004). However, since the beginning of this century, many studies have reported macrolide-resistant *M. pneumoniae* (MRMP) worldwide (Waites et al., 2017). In China, MRMP is very common, with a prevalence of 83% to 95%. Patients infected with MRMP usually respond poorly to macrolides, and exhibit more extensive lung solidification or necrosis, pleural effusion, and longer periods of fever and hospitalization (Yen et al., 2023).

Therefore, it is necessary to monitor the prevalence of MRMP and raise a warning of increased MRMP prevalence. Due to the difficulty in isolating and cultivating of *M. pneumoniae*, only a few reports on using minimum inhibitory concentrations (MICs) to evaluate the antibiotic sensitivity of *M. pneumoniae* isolates can be found (Wu et al., 2020; Xiao et al., 2020; Burgos et al., 2023). In this study, we collected and cultured isolates from children hospitalized with *M. pneumoniae* pneumonia (MPP) at the Children’s Hospital affiliated to Capital Institute of Pediatrics, Beijing, China, from 2021 to 2023. We selected six antibiotics to determine the MICs of *M. pneumoniae* isolates, analyzed molecular characteristics including P1 typing and multi-locus variable-number tandem-repeat analysis (MLVA), and macrolide resistance associated mutations. We also collected patient information, and analyzed the clinical features. We intended to identify the reasons for the resurgence of *M. pneumoniae* outbreaks and high severity rate of pneumonia in China in 2023.

## 2 Materials and methods

### 2.1 Ethics statement

This study was performed in compliance with the Ethical Principles for Medical Research Involving Human Subjects of the Declaration of Helsinki, and was approved by the research board of the Ethics Committee of the Capital Institute of Pediatrics in Beijing, China (SHERLL2023092). Informed consent was obtained during admission for patient clinical records and specimens to be collected. Data was accessed anonymously and used in statistical analysis.

### 2.2 Clinical data

In this study, we retrospectively analyzed 62 pediatric cases diagnosed with MPP and hospitalized in the Children’s Hospital affiliated to Capital Institute of Pediatrics in Beijing, China, between January 1, 2021 and December 31, 2023. For each case, the clinical presentation and laboratory detection data were recorded. Severe *M. pneumoniae* pneumonia (SMPP) was diagnosed on the basis of fever (>39°C) ≥5 days or fever ≥7 days, radiological deterioration or consolidation present in >2/3 of the lung lobes, and intra‐ and extrapulmonary complications, according to the National Health Commission’s ‘Guidelines for the Diagnosis and Treatment of *M. pneumoniae* in Children (2023 edition)’ (Yan et al., 2019; Commission, 2023).

### 2.3 *M. pneumoniae* culture and quantification

All isolates were obtained from sputum or bronchoalveolar lavage fluid collected from MPP patients. The specimens were obtained and promptly transported to the bacterial laboratory and *M. pneumoniae* was detected using the nucleic acid and resistance mutation site detection kit (Jiangsu Mole BioScience Co., Ltd), with positive ones were selected for culturing (Dorigo-Zetsma et al., 1999). After centrifugation (12000rpm, 5min), the DNA of *M. pneumoniae* was extracted using the Bacteria Genomic DNA Kit (Tiangen, YDP302). The Real-time PCR mixture was prepared in a total volume of 25 μl, containing 5 μl of sample DNA. Real-time PCR was performed under the following conditions: initial activation at 95°C for 30s, followed by 40 cycles at 95°C for 5 s and 63°C for 30s. According to the quantitative result, the concentration of *M. pneumoniae* was adjusted to 10^4^–10^5^ colony-forming units for experimental use.

### 2.4 Antimicrobial susceptibility testing

Following the guidelines of the Clinical and Laboratory Standards Institute (CLSI) M43-A (2011 edition) (Waites et al., 2011), six antibiotics (erythromycin, azithromycin, levofloxacin, chloramphenicol, acetylspiramycin, and tetracycline) were dissolved and diluted, and two-fold serial dilutions ranging from 1024 µg/mL to a minimum of 0.125 µg/mL were conducted. The MIC of each antibiotic was determined using the broth microdilution method. In each well of a 96-well plate, 100 µL of the *M. pneumoniae* dilution and 100 µL of the antibiotic was added. The positive control strain was *M. pneumoniae* M129 (ATCC29342), along with drug dilution and medium as the negative control. The plates were placed in a CO_2_ incubator at 37°C for 4-6 days until the positive control well first showed color change. Each strain was tested in triplicates.

### 2.5 Molecular characteristics analysis

For analysis of molecular characteristics of *M. pneumoniae*, the genomic DNA of each isolate was extracted. Subsequently, polymerase chain reaction restriction fragment length polymorphism analysis (PCR-RFLP) was used to analyze the P1 genotypes. Products of type 2 specimens were sequenced to identify type 2 variants (Sun et al., 2013). Variable‐number tandem-repeat loci (Mpn13, Mpn14, Mpn15, and Mpn16) were amplified in a single reaction using a multiplex PCR‐capillary electrophoresis assay (Dumke and Jacobs, 2011; Yan et al., 2019). This approach facilitated the differentiation of various repetitive sequences derived from the *M. pneumoniae* P1 gene. Furthermore, the sequence of the V domain of the 23S rRNA gene associated with macrolide resistance was amplified and sequenced. All primers synthesis and products sequencing were completed by Biotech (Shanghai) Co., Ltd.

### 2.6 Detection of co-infection

Respiratory Pathogens Nucleic Acid Detection Kit (CapitalBio Technology Co., Ltd.) was used to detect *Streptococcus pneumoniae*, *Staphylococcus aureus*, *Methicillin-resistant Staphylococcus*, *Klebsiella pneumoniae*, *Haemophilus influenzae*, *Pseudomonas aeruginosa*, *Acinetobacter baumannii* and *Serratia marcescens*. Resp®13 Respiratory Pathogen Multiplex Kit (Ningbo Health Gene Technology Co., LTD.) was used to detect Chlamydia pneumoniae and respiratory viruses. The specimens were inoculated on Sabouraud’s AGAR medium and cultured at 37°C and 5% CO_2_ for 48h for fungal identification.

### 2.7 Statistical analysis

The collected data were analyzed using SPSS Version 27.0, employing the chi-square test for categorical variables and the rank sum test for rank variables. A *p* value < 0.05 was considered to indicate statistical significance.

## 3 Results

### 3.1 Antimicrobial susceptibility analysis of *M. pneumoniae* isolates

**Figure 1:**
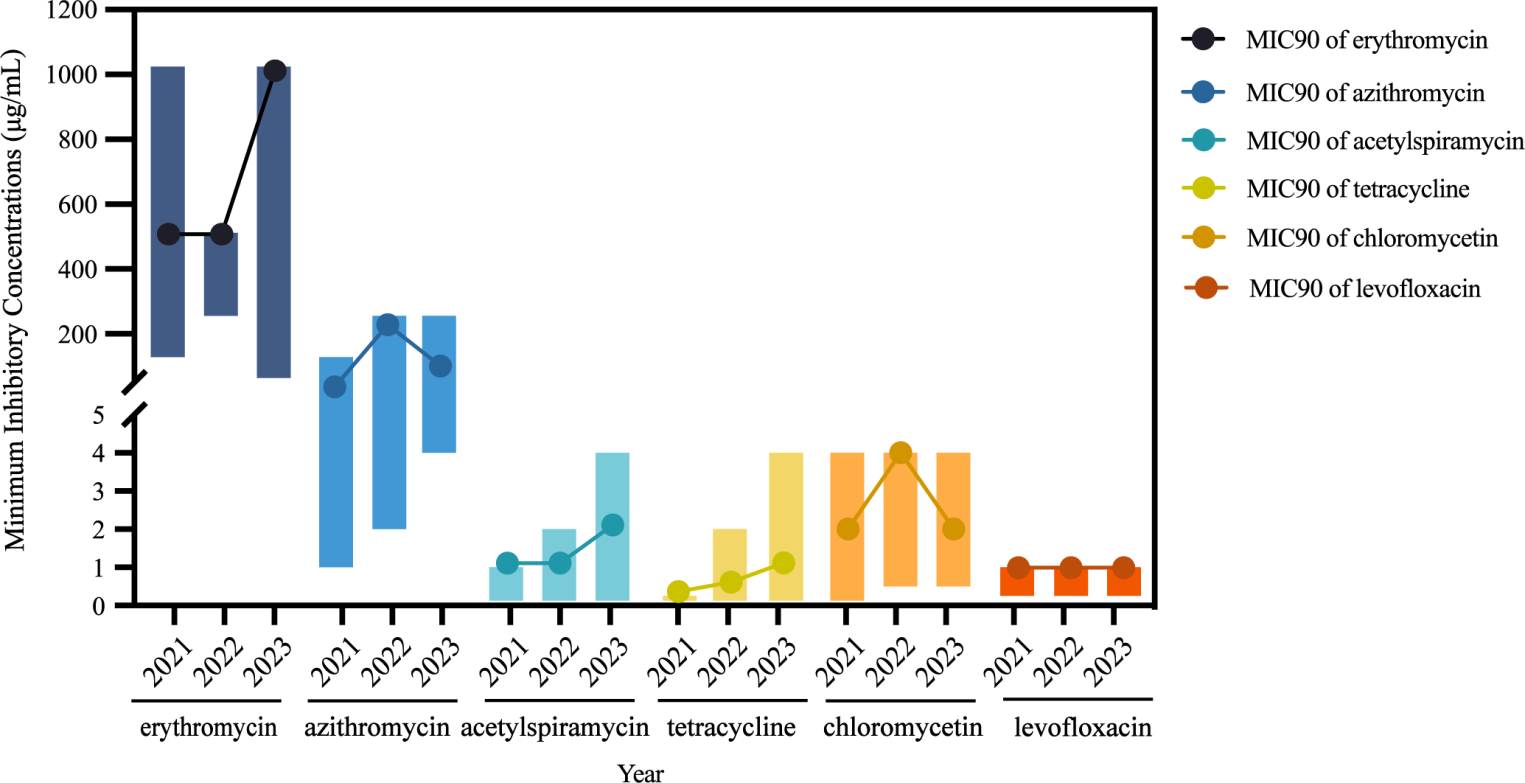
Variation of minimum inhibitory concentrations of *Mycoplasma pneumoniae* isolates.

Sixty-two *M. pneumoniae* isolates collected from the years 2021 (13/62), 2022 (18/62), and 2023 (31/62) were tested. Notably, all isolates exhibited resistance to erythromycin (14-membered macrolides) and azithromycin (15-membered macrolides). The MICs of erythromycin ranged from 64 μg/mL to 1024 μg/mL, while that for azithromycin ranged from 1 μg/mL to 256 μg/mL. A comparative analysis of the MICs of azithromycin across the three years revealed a significant increase over time (*p* < 0.001). Specifically, the MICs of azithromycin in 2023 was notably higher than those in 2021 (*p* = 0.002) and 2022 (*p* = 0.025). Furthermore, the MIC_50_ of azithromycin rose from 16 μg/mL in 2021 to 64 μg/mL in 2023, while the MIC_90_ increased from 64 μg/mL to 128 μg/mL. Similarly, the MIC_90_ of erythromycin exhibited an upward trend from 512 μg/mL in 2021 to 1024 μg/mL in 2023. Acetylspiramycin (16-membered macrolides) displayed a lower MIC range of <4 μg/mL, with MIC_50_ and MIC_90_ values of 0.25 μg/mL and 1 μg/mL, respectively. Over the years, the MIC_50_ of acetylspiramycin increased from <0.125 μg/mL in 2021 to 0.5 μg/mL in 2023, while the MIC_90_ rose from 1 μg/mL to 2 μg/mL, consistent with the increasing trend of erythromycin and azithromycin (**Figure 1**).

All isolates, including the reference strain M129, demonstrated susceptibility to tetracycline, with MIC ranging from <0.125 μg/mL to 1 μg/mL. Moreover, all isolates are sensitive to levofloxacin, with MIC ranging from <0.25 μg/mL to 1 μg/mL. Chloramphenicol displayed a MIC range of <0.125–4 μg/mL, with MIC_50_ and MIC_90_ values of 2 μg/mL and 4 μg/mL, respectively (**Table 1**).

**Table 1:** Minimum inhibitory concentrations of six antimicrobial drugs against 62 *Mycoplasma pneumoniae* Isolates.

Antibiotics	Number of resistant isolates			Total number of drug-resistant isolates	Overall resistance rate	MIC^a^ range (μg/mL)	M129 (μg/mL)	MIC_50_ ^b^ (μg/mL)	MIC_90_ ^c^ (μg/mL)	Interpretive Criteria	
	2021	2022	2023							S	R
Erythromycin	13/13	18/18	31/31	62/62	100%	64-1024	<0.125	512	512	≤0.5	≥1
Azithromycin	13/13	18/18	31/31	62/62	100%	1-256	<0.125	32	128	≤0.5	≥1
Acetylspiramycin	–	–	–	–	–	<0.125-4	<0.125	0.25	1	–	–
Tetracycline	0/13	0/18	0/31	0/62	0	<0.125-1	<0.125	0.25	0.5	≤2	–
Chloromycetin	–	–	–	–	–	<0.125-4	2	2	4	–	–
Levofloxacin	0/13	0/18	0/31	0/62	0	<0.125-1	0.25	0.5	1	≤1	–

a MIC: minimum inhibitory concentration.

b MIC_50_: 50% minimum inhibitory concentration.

c MIC_90_: 90% minimum inhibitory concentration.

### 3.2 Molecular characteristics of *M. pneumoniae* isolates

**Figure 2:**
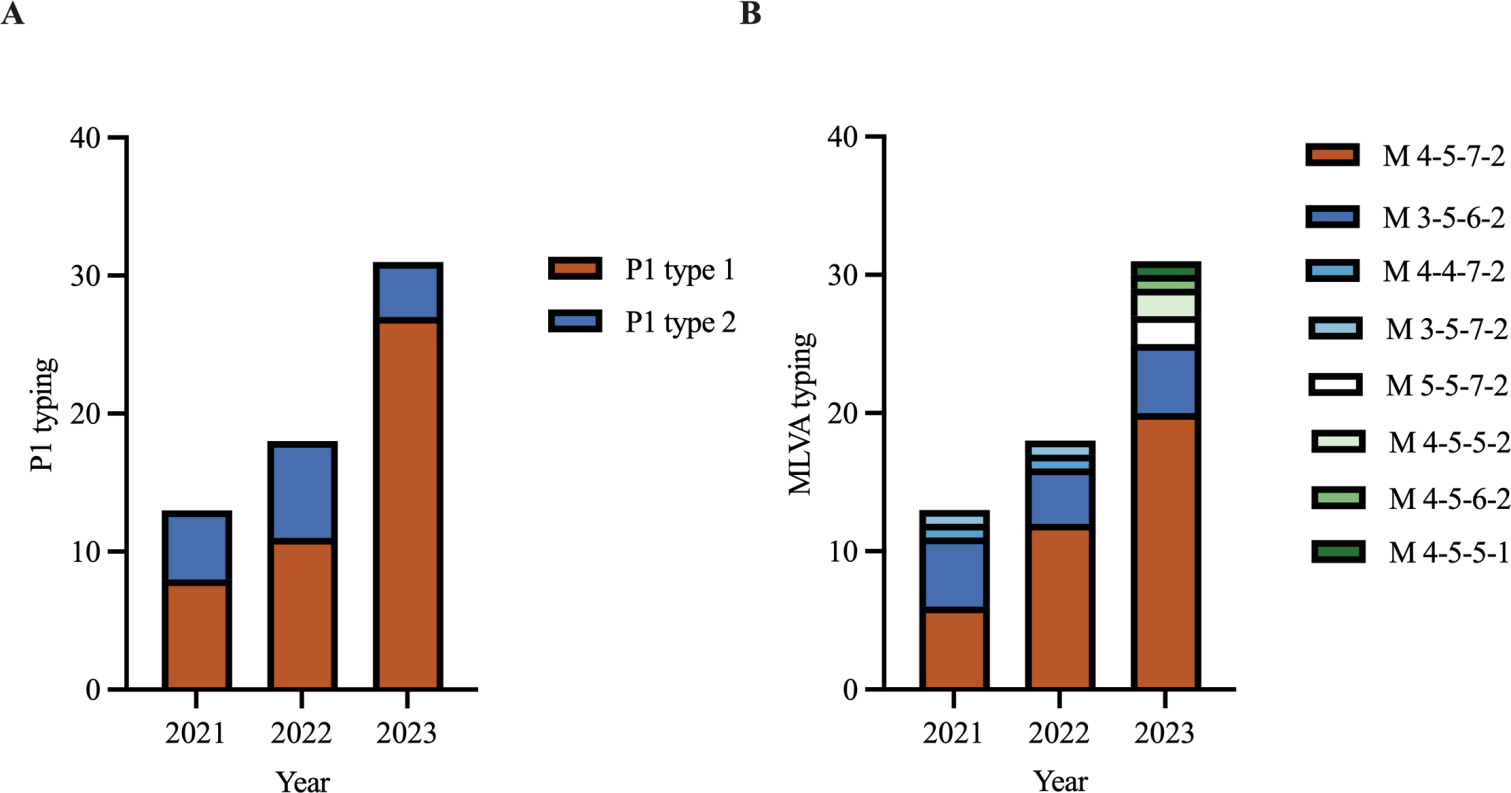
P1 typing and MLVA typing of *Mycoplasma pneumoniae* in 2021‐2023. **(A)** P1 typing of isolates in 2021‐2023. **(B)** MLVA typing of isolates in 2021‐2023.

Among the 62 isolates, 74.2% (46/62) were classified as P1 type 1 and 25.8% (16/62) were P1 type 2. When comparing the prevalence of P1 type 1 isolates across different years, we observed a significant increase in 2023 compared with that in 2021 and 2022 (*p* < 0.001) (**Figure 2A**). The MIC of erythromycin ranged from 64 μg/mL to 1024 μg/mL in type 1 isolates and ranged from 256 μg/mL to 1024 μg/mL in type 2 isolates. The MIC for azithromycin ranged from 1 μg/mL to 256 μg/mL in type 1 isolates and ranged from 2 μg/mL to 256 μg/mL in type 2 isolates. Eight distinct MLVA types were identified: M4-5-7-2 (61.3%, 38/62), M3-5-6-2 (22.6%, 14/62), M4-4-7-2 (3.2%, 2/62), M3-5-7-2 (3.2%, 2/62), M5-5-7-2 (3.2%, 2/62), M4-5-5-2 (3.2%, 2/62), M4-5-6-2 (1.6%, 1/62), and M4-5-5-1 (1.6%, 1/62) (**Figure 2B**). M4-5-7-2 and M3-5-6-2 were the main types. Additionally, 80.4% (37/46) of the P1 type 1 isolates exhibited MLVA type M4-5-7-2, whereas 75% (12/16) of the type 2 isolates displayed MLVA type M3-5-6-2. The MIC of erythromycin ranged from 64 μg/mL to 1024 μg/mL in M4-5-7-2 isolates and ranged from 256 μg/mL to 1024 μg/mL in M3-5-6-2 isolates. The MIC of azithromycin ranged from 1 μg/mL to 256 μg/mL in M4-5-7-2 isolates and ranged from 4 μg/mL to 256 μg/mL in M3-5-6-2 isolates (**Table 2**). All isolates possessed the A2063G mutation within the V domain of the 23S rRNA gene, mutations at site 2064, 2611 and 2617 were not detected. All clinical isolates collected in this study were MRMP.

**Table 2:** Correlation between genotypes and MICs distribution of *Mycoplasma pneumoniae* Isolates.

Characteristics	Total no. (%)	No. collected in 2021	No. collected in 2022	No. collected in 2023	MIC range of erythromycin (μg/mL)	MIC range of azithromycin (μg/mL)	MIC_50_/ MIC_90_ of erythromycin (μg/mL)	MIC_50_/ MIC_90_ of azithromycin (μg/mL)
P1 typing								
Type 1	46(74.2%)	8(61.5%)	11(61.1%)	27(87.1%)	64-1024	1-256	512/512	32/128
Type 2	16(25.8%)	5(38.5%)	7(38.9%)	4(12.9%)	256-1024	2-256	512/1024	16/128
MLVA typing								
M4-5-7-2	38(61.3%)	6(46.2%)	12(66.7%)	20(64.5%)	64-1024	1-256	512/512	32/128
M3-5-6-2	14(22.6%)	5(38.5%)	4(22.2%)	5(16.1%)	256-1024	4-256	512/1024	32/128
M4-4-7-2	2(3.2%)	1(7.7%)	1(5.6%)	0(0)	–	–	–	–
M3-5-7-2	2(3.2%)	1(7.7%)	1(5.6%)	0(0)	–	–	–	–
M5-5-7-2	2(3.2%)	0(0)	0(0)	2(6.5%)	–	–	–	–
M4-5-5-2	2(3.2%)	0(0)	0(0)	2(6.5%)	–	–	–	–
M4-5-6-2	1(1.6%)	0(0)	0(0)	1(3.2%)				
M4-5-5-1	1(1.6%)	0(0)	0(0)	1(3.2%)	–	–	–	–

### 3.3 Clinical characteristics of *M. pneumoniae* pneumonia patients

Among the 62 *M. pneumoniae* pneumonia patients, complete clinical information was available for 59 cases. Of them, 76.3% (45/59) cases exhibited mild symptoms, while 23.7% (14/59) cases were SMPP. Among the 26 cases with *M. pneumoniae* mono-infection, 19.2% (5/26) cases were SMPP and 80.8% (21/26) were general *M. pneumoniae* pneumonia (GMPP) (80.8%, 21/26). The MIC range of erythromycin against isolates from SMPP patients was 256-1024 μg/mL, and that of azithromycin was 16-256 μg/mL. For *M. pneumoniae* isolates from GMPP patients, the MIC range of erythromycin was 64-1024 μg/mL and that of azithromycin was 4-256 μg/mL. The MIC of *M. pneumoniae* isolates obtained from SMPP patients was higher than that from GMPP patients, but statistical analysis revealed no significant difference.

Thirty-three patients (55.9%) were co-infected with bacteria or viruses. Among them, *Streptococcus pneumoniae* (13.5%) and *Haemophilus influenzae* (13.5%) are the most prevalent co-pathogens, followed by *Epstein–Barr virus* (10.2%). *Parainfluenza virus*, *adenovirus*, *rhinovirus*, and *coxsackievirus* accounted for 5.1%, while *cytomegalovirus* accounted for 3.4%, and methicillin-resistant *S. aureus*, *Pseudomonas aeruginosa*, *Acinetobacter baumannii*, *Klebsiella pneumoniae*, *Serratia marcescens*, influenza virus, and oral *Candida* accounted for only 1.7%. However, there was no statistically significant disparity between the MICs of erythromycin and azithromycin and co-infection.

In MPP patients, fever lasted on an average of 12 days (1-30 days), the duration of fever after initiation treatment of macrolide antibiotics was 8 days (1-22 days), and the hospitalization time was 7 days (2-20 days). Seven patients did not feel better after the macrolide antibiotic was used and they were switched to the usage of levofloxacin, doxycycline, or cephalosporin.

## 4 Discussion

This study reported the *in vitro* antimicrobial susceptibility of *M. pneumoniae* isolates to six antibiotics and explored the molecular and clinical characteristics of these isolates obtained from the Children’s Hospital affiliated to Capital Institute of Pediatrics in Beijing, China, from 2021 to 2023. These antibiotics can be categorized into as four classes. Among them, erythromycin, azithromycin, and acetylspiramycin belong to 14-membered, 15-membered, and 16-membered macrolides, respectively, which are the first-line drugs for treating *M. pneumoniae* infection (Lee et al., 2018c; Metlay et al., 2019; Olson and Davis, 2020). Levofloxacin belongs to quinolone antibiotics, chloramphenicol belongs to chloramphenicol antibiotics, and tetracycline belongs to tetracycline antibiotics. The reference strain of *M. pneumoniae* M129, was selected as the control strain during the antimicrobial susceptibility testing. The results revealed that M129 displayed sensitivity to macrolides and tetracyclines, exhibiting MIC values of less than 0.125 μg/mL. The MICs of levofloxacin and chloramphenicol were recorded as 0.25 μg/mL and 2 μg/mL, respectively. Our study marks the report of erythromycin and azithromycin resistance reaching 100% (62/62), accompanied by a 100% (62/62) A2063G mutation.

At present, there are limited reports on the *in vitro* antimicrobial susceptibility of *M. pneumoniae*. In previous study, 65.4% of *M. pneumoniae* isolates in Beijing exhibited resistance to erythromycin and azithromycin, while maintaining susceptibility to levofloxacin and tetracycline in 2014–2016 in China (Zhao et al., 2019b). In Weihai, Shandong Province, China, the macrolide resistance rate reached 98.8% in 2019 (Guo et al., 2022). Similarly, in Japan, a significant increase in the MICs of macrolides has been reported (Ishiguro et al., 2016; Oishi et al., 2022).

Since *M. pneumoniae* was recognized and named in 1962, the antibiotic resistance rate of *M. pneumoniae* is increasing (Chanock, 1963). Mutations occurring on the peptidyl transferase loop of the 23S rRNA gene, particularly at loci 2063 or 2064, have rendered *M. pneumoniae* less susceptible to macrolide antibiotics than earlier strains (Lucier et al., 1995). In 2022, a systematic review and meta-analysis revealed the global prevalence of MRMP infections, with the highest rates observed in East Asia, reaching 53.4% (Kim et al., 2022). Over the past two decades, Korea has encountered five outbreaks of *M. pneumoniae* with a surge in macrolide resistance rate from 0% to 84.4% (Lee et al., 2018b). Similarly, in Japan in recent years, a rise in macrolide resistance rates from 53.7% to 62.3% was observed (Ishiguro et al., 2016; Oishi et al., 2022). Moreover, the macrolides resistance rate among *M. pneumoniae* isolates in various regions of China was around 79.9% during 2017–2018, with 66.7% in Beijing (Zhao et al., 2019a). In late 2023, the mutation rate of macrolide-resistant genes in 23S rRNA was up to 97.1% in Beijing (Yan et al., 2024). In this study, all of the *M. pneumoniae* isolates presented to be A2063G mutation, this rate was higher than previous reports. Although our results showed a 100% *in vitro* resistance rate to azithromycin and a 100% mutation rate at A2063G site, only seven patients switched to the usage of other antibiotics instead of macrolide antibiotics, which shows that the majority of children were effectively treated with azithromycin. It may be related to the metabolic time of azithromycin. In children, the metabolism of azithromycin is slow and the clearance rate is low (Neu, 1991; Southern and Barker, 2004; Zhang et al., 2024). After one or two courses of treatment, the concentration of azithromycin can be maintained at a relatively high level which can inhibit *M. pneumoniae* (Matzneller et al., 2013; Dekyi et al., 2024). In addition, efficacy of macrolide drugs is related to each individual’s immune level (Zheng et al., 2018).

The genotype transition from type 1 to type 2 initiated early. Between 2003 and 2016, the proportion of *M. pneumoniae* P1 type 2 escalated from 17.4% to 39.4%, and reached 54.5% in 2019 in Beijing (Zhao et al., 2015; Sun et al., 2017; Zhao et al., 2019a, 2019b). The macrolide resistance rate among P1 type 1 *M. pneumoniae* remains considerable, but this rate for type P1 type 2 has been observed to be increasing (Zhao et al., 2015, 2019a). In the current study, wherein the proportion of type P1 type 1 isolates collected in 2023 markedly exceeded that of the previous two years (*p* < 0.001). *M. pneumoniae* strains with MLVA type M3-5-6-2 and P1 type 2 were not correlated with drug resistance (Qu et al., 2013; Kenri et al., 2020). However, recent findings reveal a significant rise in macrolide resistance among strains typed M3-5-6-2 (Wang et al., 2021). In particular, we indicated a 100% resistance rate for erythromycin and azithromycin and 100% A2063G mutation rate among strains type M3-5-6-2 and P1 type 2, potentially contributing to the resurgence of *M. pneumoniae* in China in 2023, which is specifically evident in the substantial rise in pediatric cases (Conroy, 2023; Gong et al., 2024). After evaluating the antibiotics sensitivity between *M. pneumoniae* type P1 type 1 and type P1 type 2, we found that the MIC range of erythromycin and azithromycin of P1 type 1 isolates (64-1024 μg/mL;1-256 μg/mL) was slightly wider than the range of P1 type 2 isolates (256-1024 μg/mL;2-256 μg/mL), the MIC_90_ of erythromycin of P1 type 1 isolates (512 μg/mL) was lower than P1 type 2 isolates (1024 μg/mL), while the MIC_50_ of azithromycin of P1 type 1 isolates (32 μg/mL) was higher than P1 type 2 isolates (16 μg/mL). Furthermore, while specific investigations have suggested that P1 type 2 *M. pneumoniae* exhibits elevated levels of CARDS toxins and greater virulence (Lluch-Senar et al., 2015), there are currently no reports on the correlation between typing and MIC.

The duration of fever in non-hospitalized *M. pneumoniae* patients was 10 days (7-13 days) in Beijing in 2010 (Cao et al., 2010), it was 7 days (2-9 days) in macrolide-resistant *M. pneumoniae* infected children in Ningbo, Zhejiang province, China, in 2019-2022 (Chen et al., 2024). Both of the fever days were shorter than the duration in this study (12 days). The duration of fever after initiation of macrolide antibiotics was 4 days (0-8 days), which was also shorter than that of 8 days (1-22 days) in the present study, suggesting that MRMP infection increases the duration of fever and the duration of macrolide antibiotic use. Once resistance to erythromycin and azithromycin emerged, the doctors are urged to choose 16-membered cyclic macrolide antibiotics and tetracyclines when warranted, particularly if patients remain febrile or chest radiographs exhibit deterioration 48–72 hours post treatment (Lee et al., 2018a). Clinically, when azithromycin treatment is ineffective and fever persists in children with *M. pneumoniae*, tetracyclines are preferred as substitutes, among which doxycycline has fewer side effects and a higher fever reduction rate than minocycline, so doxycycline is preferred. Quinolones may also be considered in children over 8 years of age. Methylprednisolone can be used when necessary in severely ill children (Song et al., 2024).

There are some limitations in our study. Firstly, we only analyzed 62 isolates, which is a limited number that may have led to data bias and may not comprehensively and objectively represent the actual situation in Beijing. In future studies, a larger sample size covering a greater number of surveillance sites is needed. Secondly, we only detected the mutation sites of *M. pneumoniae* relevant to macrolide antibiotics in 23S rRNA gene, mutations in other sites were not included. Thirdly, the period from 2021 to 2023 is the pandemic period of COVID-19, and the *M. pneumoniae* isolates resistance data of several years before and after the COVID-19 epidemic will be added for comparison in next study.

In conclusion, this study analyzed MICs distribution of 62 *M. pneumoniae* isolates collected in Beijing from 2021 to 2023. The MIC of azithromycin in 2023 was notably higher than those in 2021 and 2022. All *M. pneumoniae* isolates possessed the A2063G mutation, conferring 100% resistance to erythromycin and azithromycin. Our findings confirm that MRMP may be the causative factor of the *M. pneumoniae* epidemic in late 2023 in Beijing, China. It is important for the doctors to pay more attention to detection of MRMP and the antibiotics choose. Acetylspiramycin, which is a 16-membered macrolide antibiotic, appears to be a promising alternative treatment, especially for resistant isolates.

## Data Availability

The original contributions presented in the study are included in the article/supplementary material. Further inquiries can be directed to the corresponding authors.
